# Function and Mechanism of Small Nucleolar RNAs (snoRNAs) and Their Host Genes (SNHGs) in Malignant Tumors

**DOI:** 10.3390/biom15111625

**Published:** 2025-11-19

**Authors:** Jiaji Yu, Yingjie Shao, Wendong Gu

**Affiliations:** 1 Department of Radiation Oncology, Changzhou Medical Center, Nanjing Medical University, 185 Juqian Street, Changzhou 213003, China; yujiaji@stu.njmu.edu.cn; 2 Department of Radiation Oncology, The First People’s Hospital of Changzhou, Changzhou 213003, China

**Keywords:** snoRNAs, SNHGs, tumors, regulatory mechanisms

## Abstract

Small nucleolar RNAs (snoRNAs) and their host genes (SNHGs) are non-coding RNAs that are integral to tumorigenesis and progression. snoRNAs contribute to tumor progression primarily through RNA modification and engagement in intracellular signaling, and by serving as precursors for small nucleolar RNA-derived RNAs (sdRNAs) that exert microRNA (miRNA)-like or epigenetic regulatory functions. SNHGs modulate key tumor cell behaviors—including proliferation, metastasis, and resistance to therapy—through competing endogenous RNA (ceRNA)-mediated interactions and epigenetic mechanisms. Their combined influence significantly impacts patient prognosis. Across diverse malignancies such as neurologic, bone, and head and neck cancers, snoRNAs and SNHGs exhibit cancer-specific regulatory dynamics; for instance, in glioblastoma, snoRNAs and their derived fragments (sdRNAs) contribute to intratumoral heterogeneity by mediating both metabolic reprogramming and epigenetic remodeling, while their mediated modulation of cellular proliferation and metastatic potential is evident in breast cancer. Concurrently, several snoRNAs and SNHGs have emerged as potential diagnostic and prognostic biomarkers, as well as therapeutic targets. Preclinical interventions targeting select snoRNAs or SNHGs have demonstrated promising therapeutic outcomes. This study reviews current insights into the oncogenic functions and signaling networks associated with dysregulated snoRNAs and SNHGs in malignancies, while highlighting novel avenues for future investigation in this domain.

## 1. Background

Small nucleolar RNAs (snoRNAs), a subclass of non-coding RNAs (ncRNAs) ranging from 60 to 300 nucleotides, function as essential guides within small nucleolar ribonucleoproteins (snoRNPs) [1]. They are categorized into three groups: C/D box snoRNAs, H/ACA box snoRNAs, and small Cajal RNAs (scaRNAs) [2]. Mammalian genomes encode approximately 400 distinct snoRNA sequences, which are indispensable for 2′-*O*-methylation, pseudouridylation, and the processing of various RNA species [3]. C/D box snoRNAs (SNORDs) direct site-specific 2′-*O*-ribose methylation of ribosomal RNA (rRNA) or small nuclear RNA (snRNA), whereas H/ACA box snoRNAs (SNORAs) mediate the conversion of uridine to pseudouridine [4]. Typically, eukaryotic C/D box snoRNAs span 70 to 120 nucleotides, while H/ACA box snoRNAs range from 60 to 75 nucleotides and possess a distinct pseudouridylation pocket critical for uracil isomerization on substrate RNAs [2]. C/D box snoRNAs mediate 2′-*O*-methylation of specific nucleotides in target RNAs by assembling with four core proteins: nucleolar protein 56 (NOP56), NOP58, SNU13, and the methyltransferase fibrillarin (FBL). In parallel, H/ACA box snoRNAs interact with conserved proteins including glycine- and arginine-rich protein 1 (GAR1), non-histone chromosomal protein 2 (NHP2), NOP10, and dyskeratosis congenita 1 (DKC1), directing the site-specific isomerization of uridine to pseudouridine [5]. scaRNAs, a distinct subclass of snoRNAs also ranging from 60 to 300 nucleotides, localize specifically to the Cajal body within the nucleus. They regulate chemical modifications of snRNAs, predominantly 2′-*O*-methylation and pseudouridylation, processes indispensable for both spliceosomal snRNA maturation and rRNA modification within the Cajal body [6,7]. By facilitating snRNA maturation, scaRNAs exert regulatory control over pre-mRNA splicing [7].

Most snoRNAs are embedded within the intronic regions of both protein-coding and non-coding genes, collectively referred to as snoRNA host genes (SNHGs). Following transcription, the primary SNHG RNA—including all exons, introns, and associated snoRNAs—is processed into distinct functional components: introns are processed to snoRNAs predominantly active in the nucleolus; exons undergo alternative splicing and localize to the cytoplasm; and the full-length transcript, comprising exon regions, is preserved as long noncoding RNAs known as lncSNHGs [8,9,10,11].

Small nucleolar RNA-derived RNAs (sdRNAs) represent a novel class of functionally active short non-coding RNAs (17–30 nt) processed from full-length snoRNAs [12]. Initially dismissed as random degradation products, sdRNAs are now recognized as stable molecules with conserved expression across species [13]. They exhibit miRNA-like properties, including the ability to associate with Argonaute (AGO) proteins and integrate into the RNA-induced silencing complex (RISC) to post-transcriptionally regulate gene expression [12]. Their biogenesis can occur via canonical miRNA pathways or non-canonical routes involving snoRNP proteins such as fibrillarin and NOP58 [13].

The abnormal expression patterns of snoRNAs and SNHGs across a spectrum of malignancies have drawn considerable interest due to their multifaceted roles in tumorigenesis or progression and their strong correlation with clinical outcomes (Table 1).

### 1.1. Classification, Biogenesis, and Non-Canonical Roles of snoRNAs

Small nucleolar RNAs (snoRNAs) are broadly classified into two principal families based on their conserved sequence motifs: the C/D box snoRNAs (SNORDs) and the H/ACA box snoRNAs (SNORAs). Canonically, SNORDs guide site-specific 2′-*O*-ribose methylation of target RNAs, primarily by assembling with core proteins like the methyltransferase fibrillarin (FBL). In contrast, SNORAs, defined by a hairpin-hinge-hairpin structure, primarily mediate the enzymatic conversion of uridine to pseudouridine (Ψ), a reaction catalyzed by the core protein Dyskerin (DKC1) [4,53].

Beyond these two main families, specialized types exist, such as small Cajal body-specific RNAs (scaRNAs), which are often hybrid molecules containing both C/D and H/ACA domains to guide modifications on spliceosomal snRNAs [6,7]. Crucially, a large fraction of snoRNAs are “orphans” with no known complementarity to rRNAs or snRNAs [54]. Their high conservation suggests they have been co-opted for non-canonical functions, such as regulating alternative splicing and gene expression, which is highly relevant to their roles in cancer [55].

The biogenesis of snoRNAs is intrinsically linked to their genomic organization and provides a key mechanism for their co-regulation in disease. The vast majority of human snoRNA sequences are embedded within the intronic regions of snoRNA host genes (SNHGs) [53]. Consequently, their production is inextricably linked to the splicing of the SNHG pre-mRNA. Following splicing and intron liberation, the mature snoRNA is processed by exonucleases [56]. This genomic arrangement ensures that the expression of snoRNAs is often coupled with their host genes, establishing a SNHG-snoRNA regulatory axis that is frequently dysregulated in malignant tumors [8,9,10,11].

### 1.2. Biogenesis and Function of sdRNAs

#### 1.2.1. Biogenesis of sdRNAs Associating with Argonaute 2 (AGO2)

Full-length snoRNAs can be generated either as products of transcription or splicing. snoRNAs produced by transcription can give rise to microRNA-like sdRNAs which are specifically excised from parent snoRNA transcripts via the classical miRNA processing pathway. This occurs by processing of parent snoRNAs into smaller transcripts by the microprocessor complex, consisting of ribonuclease III DROSHA and DiGeorge syndrome critical region 8 protein (DGCR8). The intermediate snoRNA transcript is then exported to the cytoplasm via exportin 5 (XPO5). Following this, the smaller snoRNA is processed in the cytoplasm by the ribonuclease III endonuclease Dicer (DICER) to generate the mature sdRNA. This sdRNA associates with AGO2 protein, leading to the formation of the RISC. Similar to miRNAs, these sdRNAs function in post-transcriptional gene suppression by antisense binding to target mRNA transcripts within RISC [13].

However, snoRNAs produced by splicing can also enter the classical miRNA processing pathway. Nevertheless, spliced snoRNAs can circumvent processing by DROSHA/DGCR8 and/or DICER because they are trafficked to the nucleolus and subsequently processed by the fibrillarin complex, followed by cytoplasmic export via a transporter belonging to the Exportin (XPO) family of proteins [13].

Research has revealed that snoRNAs can serve as important sources of functional small RNAs (such as miRNAs) through alternative biogenesis pathways that operate independently of the canonical enzyme Dicer. This mechanism may have global significance in malignancies: a study on colon cancer found that under Dicer-deficient conditions, the overall gene regulatory program of cells can shift from traditional Dicer-dependent miRNA expression towards Dicer-independent expression of sdRNAs. This provides a new perspective for understanding the pathogenesis of Dicer-related human diseases [57].

This non-canonical biogenesis pathway is corroborated at the molecular level. For instance, the atypical box C/D snoRNA U3, which participates in ribosome assembly, can be processed from a specific stem-loop structure in its 5′ domain into short fragments that associate with Argonaute proteins, forming “miR-U3.” This processing is independent of both Drosha and Dicer, indicating that the full-length U3 snoRNA can be exported to the cytoplasm for processing. Functional assays have confirmed that miR-U3 can act as a low-proficiency miRNA in vivo, regulating endogenous targets such as SNX27 mRNA. Most importantly, perturbation of U3 snoRNP assembly itself induces the production of miR-U3, suggesting a potential novel cross-regulatory mechanism between ribosome biogenesis and gene expression [58].

#### 1.2.2. Biogenesis of PIWI-Associated RNA (piRNA)-like sdRNAs

Spliced snoRNAs that enter the nucleolus for fibrillarin processing can be trafficked to Yb bodies via Nuclear RNA Export Factor (NXF1)/Nuclear Transport Factor 2-like Export Factor 1 (NXT1). Within the Yb bodies, their 3′ end is cleaved by Zucchini (ZUC) and subsequently degraded. The remaining transcript is further processed within the Yb body by the papi-dependent trimmer. Subsequently, the HEN1 double-stranded RNA-binding protein adds a methyl group to the 3′ end of the transcript, generating a mature piRNA/PIWI complex. This complex is then exported to the cytoplasm. Subsequently, this piRNA/PIWI complex can be shuttled back into the nucleus, where it functions to inhibit transcription [13].

### 1.3. A Compendium of Common Human Small Nucleolar RNAs

The expanding research into snoRNAs has identified hundreds of distinct molecules, each with specific functions and, increasingly, links to human disease. While a comprehensive catalog is beyond the scope of this report, an examination of several well-characterized examples illustrates the remarkable functional breadth of this class of RNAs. The following table synthesizes data from numerous publications and specialized databases to provide a high-value, quick-reference summary of key human snoRNAs, juxtaposing their canonical roles with emerging non-canonical functions and disease associations [59] (Table 2).

## 2. In-Depth Elaboration on Key Examples

The roles of snoRNAs in cancer are not monolithic; they can function as potent oncogenes or as critical tumor suppressors, depending on the specific snoRNA and the cellular context. This duality is perfectly illustrated by comparing SNORA42 and SNORD44.

SNORA42 has emerged as a clear-cut oncogene. It is consistently upregulated in multiple aggressive malignancies, including non-small-cell lung cancer (NSCLC), hepatocellular carcinoma (HCC), and colorectal cancer (CRC), where its high expression is correlated with poor patient prognosis. Functionally, SNORA42 promotes cancer cell proliferation, migration, and invasion while inhibiting apoptosis. Mechanistic studies have revealed that it exerts these effects by modulating core cancer signaling pathways. For example, it can inhibit the tumor-suppressive p53 pathway and activate the pro-survival PI3K/Akt pathway, thereby tilting the cellular balance toward malignant transformation [60].

In contrast to SNORA42, SNORD44 may exhibit context-dependent roles. For instance, in prostate cancer, SNORD44 and its derived sdRNAs are upregulated during malignant transformation. Further research is needed to clarify its dual functions [61]. Functionally, SNORD44 acts in concert with its host gene to suppress cell proliferation and induce apoptosis. Overexpression of SNORD44, for instance using an oncolytic adenovirus, can inhibit tumor growth both in vitro and in vivo [62].

These examples demonstrate that snoRNAs are integral components of the cell’s regulatory circuitry. They are not simply passive bystanders whose levels change during tumorigenesis; they are active participants that can be co-opted by cancer cells (like SNORA42) or whose loss can remove a critical brake on proliferation (like SNORD44). The even more complex case of SNORA21, which acts as an oncogene in gastric cancer but a tumor suppressor in gallbladder cancer, underscores the profound context-dependency of snoRNA function, likely dictated by the unique molecular wiring of different cell types [63,64].

### 2.1. Functions of snoRNAs and SNHGs in Various Types of Malignancies

#### 2.1.1. Neurologic Malignancies

1. Glioblastoma

In recent years, the regulatory functions of snoRNAs and SNHGs in glioblastoma (GBM) have been increasingly elucidated. U3 snoRNA is transcriptionally activated via promoter hypomethylation, generating sdRNA fragments that target ZBTB7A, thereby relieving its repression of glycolytic enzymes HK2 and LDHA. This shift enhances aerobic glycolysis and proliferative capacity in IDH1 wild-type GBM [14]. Additionally, U3 snoRNA cooperates with TRIM24 to recruit DNA-PKcs, triggering phosphorylation events that induce epigenetic remodeling and promote the epithelioid transformation of GBM [15]. While this study [15] investigated the full-length U3 snoRNA, it is important to note that U3-derived sdRNAs have also been shown to mediate epigenetic silencing [65]. It is plausible that both the metabolic and epigenetic remodeling functions attributed to U3 in GBM are, in fact, executed by its derived sdRNA fragments, highlighting a critical non-canonical pathway. Elevated levels of snoRNAs such as SNORD88C have been associated with unfavorable prognosis, accelerating the malignant progression of low-grade glioma (LGG) by modulating RNA splicing and altering the immune microenvironment [66]. The lncRNA SNHG12, transcriptionally upregulated through DNA hypomethylation, functions as a competing endogenous RNA (ceRNA) for miR-129-5p, enhancing MAPK1 and E2F7 expression, which activates the MAPK/ERK cascade and drives cell cycle progression, contributing to temozolomide resistance. Molecular subclassification based on SNHG expression (SNHGscore) has revealed that GBMs with high scores predominantly exhibited mesenchymal characteristics, marked by suppressed immune infiltration, diminished responsiveness to anti-PD-1/L1 therapy, and heightened sensitivity to EGFR/ERK-MAPK-targeted agents [16,67]. Collectively, snoRNAs and SNHGs integrate epigenetic regulation, metabolic remodeling, and signal transduction to shape GBM heterogeneity and therapeutic resistance, offering a novel framework for targeted intervention.

2. Neuroblastoma

Recent evidence implicates snoRNAs and SNHGs in the initiation, drug resistance, and epigenetic modulation of neuroblastoma (NB). SNHG7 acts as a molecular sponge for miR-323a-5p and miR-342-5p, thereby derepressing CCND1 and enhancing cellular migration, invasion, and glycolytic activity [17]. SNHG16 elevates PLK4 expression via a miR-338-3p-dependent ceRNA axis, activating the PI3K/AKT pathway and contributing to cisplatin resistance [18]. Moreover, SNHG1 modulates chromatin accessibility through interactions with HDAC1/2, sustaining the transcriptional landscape characteristic of MYCN-amplified NB. Concurrently, SNHG25 recruits DKC1 to stabilize SNORA50C, suppresses HDAC1 ubiquitination and proteasomal degradation, and promotes tumor cell proliferation. SNORA50C, in turn, enhances NB cell growth and migration by preserving HDAC1 stability in an SNHG25-dependent manner [68,69]. Collectively, SNHGs drive NB progression via ceRNA interactions, epigenetic remodeling, and regulation of protein homeostasis, with the synergistic actions between snoRNAs and host genes further intensifying oncogenic potential and indicating a novel axis for targeted therapeutic development.

#### 2.1.2. Bone Malignancies

The regulatory functions of snoRNAs and SNHGs in bone malignancies have received increasing attention. SNHG6 has been identified as a scaffold for histone methyltransferase EZH2, promoting H3K27me3-mediated silencing of the tumor suppressor gene KLF6 and establishing a positive feedback loop with transcription factor SP1, thereby promoting chondrosarcoma cell proliferation, migration, and invasion [19]. Moreover, transcriptomic analysis revealed 71 upregulated and 117 downregulated snoRNAs in osteosarcoma cells; several of these differentially expressed snoRNAs modulated tumor progression by suppressing cellular proliferation and migration [70]. Collectively, SNHGs appear to advance chondrosarcoma via epigenetic remodeling, while dysregulated snoRNA expression may offer diagnostic or therapeutic utility in osteosarcoma.

#### 2.1.3. Head and Neck Squamous Cell Carcinoma (HNSCC)

Their regulatory functions of snoRNAs and SNHGs in head and neck squamous cell carcinoma (HNSCC) have become increasingly evident. A machine learning–based analysis identified five snoRNAs, subsequently integrated into a prognostic characteristic model exhibiting high sensitivity and specificity in predicting patient survival. This signature was also implicated in modulating malignant phenotypes and DNA/RNA editing processes, offering promising biomarkers for stratified HNSCC management [71]. Host genes such as SNHG3 interact closely with ribosomal proteins, including RPS27A and RPL8, through m(5)C RNA modifications, thereby influencing ribosome biogenesis, metabolic remodeling, and immune regulation, ultimately promoting HNSCC progression [20]. These observations suggest snoRNAs function independently as prognostic indicators, while SNHGs exert influence on the tumor microenvironment (TME) via epigenetic pathways and ribosomal activity. Collectively, they represent potential therapeutic targets for precision intervention in HNSCC.

#### 2.1.4. Digestive System Malignancies

1. Esophageal cancer

In esophageal squamous cell carcinoma (ESCC), snoRNAs and SNHGs contribute to tumor progression via distinct molecular pathways. SNHG12 can enhance BMI1 expression by sponging miR-6835-3p and recruiting IGF2BP2 to stabilize CTNNB1 mRNA, thereby inducing epithelial–mesenchymal transition (EMT), reinforcing stem-like properties, and accelerating metastasis. Acting as a ceRNA, SNHG17 binds miR-338-3p, relieving its suppressive effect on SOX4 and consequently promoting cellular proliferation and invasion [21,22]. SNORD12B markedly promotes ESCC cell growth, migration, and metastatic capacity by disrupting the nuclear-cytoplasmic distribution of PP-1α and activating the AKT-mTOR-4EBP1 signaling cascade [72]. Collectively, SNHGs amplify oncogenic activity through ceRNA interactions and post-transcriptional regulation, whereas snoRNAs drive tumor progression by regulating critical signaling networks. Both delineate promising avenues for targeted therapeutic strategies in ESCC.

2. Gastric cancer

In gastric cancer (GC), SNHGs, as lncRNAs, drive tumor progression via several mechanisms. SNHG12 relieves YWHAZ inhibition by sponging miR-218-5p, thereby activating the β-catenin signaling pathway to promote metastasis and EMT. Additionally, SNHG12 can be transported via exosomes, leading to the upregulation of E2F7 and subsequent activation of the MAPK/ERK pathway, which induces peritoneal mesothelial cell apoptosis and mesenchymal transition [23,24]. SNHG22 binds to EZH2, repressing tumor suppressor genes, while upregulating Notch1 through the adsorption of miR-200c-3p, thereby enhancing cell proliferation and invasion in GC [25]. As a ceRNA, SNHG1 targets miR-195-5p, leading to the upregulation of YAP1, activation of the Hippo pathway, and promotion of tumor growth [73]. SNHG26 interacts with nucleolin (NCL) to establish a positive feedback loop in energy metabolism through the c-Myc/HK2 axis, driving metastasis [74]. In conclusion, SNHGs promote GC progression via ceRNA interactions, signaling pathways, and metabolic regulation, presenting new avenues for targeted therapies.

3. Colorectal cancer

In colorectal cancer, snoRNAs and SNHGs contribute to tumor progression through various molecular mechanisms. SNORA24 promotes cell proliferation and tumor growth by destabilizing the p53 protein via the proteasome pathway. SNORA56 enhances the translation of the glutamate-cysteine ligase catalytic subunit by mediating pseudouridylation at the U1664 site of 28S rRNA, thereby inhibiting ferroptosis and accelerating tumor progression. SNORA28 recruits BRD4 to the LIFR promoter region, increasing H3K9 acetylation and activating the JAK1/STAT3 pathway, which in turn enhances colorectal cancer cell radioresistance. The SNORD12C/78 and ZFAS1-NOP58 axis synergistically regulate 2′-*O*-methylation of ribosomal RNA, promoting tumorigenesis [26,75,76,77]. Additionally, snoRNAs such as SNORA51 are markedly upregulated in fecal samples, indicating their potential as non-invasive diagnostic markers [78]. Regarding SNHGs, SNHG17 upregulates CXCL12-mediated angiogenesis by sponging miR-23a-3p and forms a positive feedback loop with the Trim23-PES1 axis and miR-339-5p-FOSL2, thereby synergistically promoting tumor proliferation and metastasis [27,28]. SNHG16 activates the YAP1/TEAD1 complex by sponging miR-195-5p, driving EMT and promoting liver metastasis [79]. SNHG1 can stabilize SERPINA3 mRNA by binding to HNRNPD, thereby enhancing metastatic potential and serving as a prospective biomarker for detecting peritoneal free cancer cells [80,81]. SNHG8 modulates autophagy and apoptosis via the AKT/AMPK/mTOR pathway, suppressing tumor progression [82]. Additionally, SNHG11 promotes hypoxia-driven metastasis by stabilizing HIF-1α. SNHG6 correlates with tumor stage and metastasis as a prognostic marker, and SNHG22 promotes cell proliferation and invasion through the miR-128-3p/E2F3 axis [83,84,85]. These studies highlight their role as promising targets for colorectal cancer diagnosis, prognosis evaluation, and targeted therapies, through mechanisms including epigenetic modification, RNA stability regulation, signaling pathway activation, and metabolic reprogramming.

4. Liver malignancies

In hepatocellular carcinoma (HCC), snoRNAs and SNHGs contribute to tumorigenesis, metastasis, and resistance to treatment by engaging in complex molecular networks with intricate regulatory mechanisms. SNORD52 promotes cell cycle progression by stabilizing CDK1 [29], while SNHG17 binds to LRPPRC, inhibiting c-Myc ubiquitination and promoting the G1/S phase transition [86]. Together, these factors drive tumor growth, while SNORA42 disrupts cell cycle regulation by targeting the p53 pathway [87], and SNORD17 inhibits p53 activity by interacting with NPM1/MYBBP1A [88]. In metabolic reprogramming, SNHG6 enhances glycolysis by stabilizing BOP1 [88], SNORA55 mediates TRPM8-induced nuclear-mitochondrial communication to regulate ATP synthase function [89], and SNORA14A suppresses succinate accumulation by upregulating SDHB, thereby slowing hepatoblastoma progression [90], emphasizing the link between metabolic imbalance and tumor energy adaptation. In epigenetics and signaling, SNHG14 activates PABPC1 via H3K27 acetylation and promotes angiogenesis through collaboration with the PTEN pathway [90], while SNHG5 regulates stemness through the UPF1/Wnt pathway [91] and cooperates with the NF2/Hippo axis to promote fibrosis [92], emphasizing the central role of epigenetic modifications and pathway interactions. Regarding the TME and drug resistance, SNHG6 impairs T cell-mediated immune responses via small extracellular vesicles [93], while SNHG16 enhances angiogenesis through the activation of the PI3K/Akt/mTOR pathway facilitated by exosome-mediated delivery [94]. Additionally, SNHG1 induces sorafenib resistance by upregulating SLC7A11 through the SND1/m6A modification [30], and SNHG19 alternative splicing contributes to chemotherapy resistance [95], collectively creating a vicious circle of immunosuppression and therapeutic failure. In clinical translation, snoRNAs like SNORD12 and SNORA47 have demonstrated prognostic significance in non-viral liver cancer [96], and SNHG6 correlates with tumor staging [88]. Targeting SNORD88B in combination with MST1 agonists [97] or intervening in the SNHG1/SND1 pathway [30] offers promising avenues for combined therapeutic strategies. In conclusion, snoRNAs and SNHGs establish a complex regulatory network that influences the cell cycle, metabolism, epigenetics, and the microenvironment. The integration of these mechanisms provides a foundation for liver cancer stratification and targeted therapeutic interventions. Future research should focus on further elucidating the dynamic regulatory networks and the clinical feasibility of these strategies.

5. Pancreatic cancer

In pancreatic cancer (PC), snoRNAs and SNHGs contribute to tumor progression and drug resistance through diverse mechanisms. Regarding SNHG regulation, the lncRNA SNHG6 relieves miR-26a-5p-mediated repression of FUBP1 by sponging the miRNA, thereby promoting EMT through upregulation of N-cadherin, vimentin, and β-catenin, while downregulating E-cadherin. This leads to enhanced proliferation, migration, and invasion of PC cells. In vivo data further support the potential of SNHG6 knockdown to suppress tumor growth, highlighting its viability as a therapeutic target [31]. In terms of snoRNA-associated mechanisms, RRP9, a protein binding to U3 snoRNA, is overexpressed in PC and activates the AKT pathway through interaction with IGF2BP1. This improves DNA repair, inhibits apoptosis, and promotes resistance to gemcitabine. Co-treatment with AKT inhibitors, such as MK-2206, can reverse the resistance phenotype [32]. From a clinical perspective, targeting SNHG6 or inhibiting the RRP9/AKT axis are promising strategies for enhancing treatment efficacy by modulating the EMT process or restoring chemotherapy sensitivity. The combined silencing of SNHG6 and inhibition of AKT could further improve therapeutic outcomes. In conclusion, SNHGs and snoRNA-related molecules elucidate a complex regulatory network of non-coding RNAs in PC, affecting core pathways such as EMT and AKT signaling, with implications for the development of targeted therapies and combination drug strategies.

#### 2.1.5. Lung Cancer

In recent years, their regulatory roles in lung cancer have become increasingly well-defined. At the molecular level, snoRNAs contribute to lung cancer progression primarily through epigenetic modifications and modulation of signaling pathways. For instance, H/ACA box snoRNAs (e.g., SNORA65, SNORA7A/B) promote non-small-cell lung cancer (NSCLC) proliferation and metastasis via ribosomal pseudouridylation [33]. Additionally, SNORD88C enhances the 2′-*O*-methylation of 28S rRNA, leading to upregulation of Stearoyl-CoA Desaturase 1, suppression of autophagy, and accelerated tumor growth [98]. Notably, serum exosomal snoRNAs, such as U78 and U37, exhibit significant overexpression in malignant lung nodules, suggesting their potential as non-invasive diagnostic markers [99]. SNHG family members regulate critical signaling pathways via the ceRNA mechanism, influencing lung cancer proliferation, drug resistance, and immune evasion. For example, SNHG11, which is upregulated in lung cancer and correlates with poor prognosis, promotes tumor progression by activating the Wnt/β-catenin pathway through two distinct mechanisms: acting as a ceRNA via the SNHG11/miR-4436a/CTNNB1 axis and by directly binding to β-catenin [34]. SNHG3, regulated by transcription factor E2F1, promotes cell proliferation and migration through the TGF-β and IL-6/JAK2/STAT3 pathways [100]. Additionally, SNHG5 accelerates tumor progression by activating the NF-κB pathway via the miR-181c-5p/CBX4 axis [101]. In the context of drug resistance, SNHG15 relieves the modulation on multidrug resistance protein 1 through the inhibition of miR-451, thereby contributing to gefitinib resistance [102]. Exosome-delivered SNHG7, on the other hand, activates the PI3K/AKT pathway by stabilizing the autophagy-related genes ATG5/ATG12 and degrading PTEN, which triggers macrophage M2 polarization and enhances resistance to docetaxel [103]. Additionally, the involvement of SNHGs in the immune microenvironment is significant: SNHG12 stabilizes immune checkpoint proteins via the HuR/PD-L1/USP8 axis, suppresses CD8^+^ T cell function, and promotes immune evasion [104]. While SNHG7 exhibits context-dependent effects in lung adenocarcinoma, its downregulation inhibits the Wnt signaling pathway through miR-181/CBX7 [105], yet exosome-derived SNHG7 exacerbates malignancy by modulating autophagy and immune suppression [103]. In conclusion, snoRNAs and SNHGs serve as central regulators in the initiation, metastasis, drug resistance, and immune escape of lung cancer through diverse mechanisms, including epigenetic modification, signal pathway interactions, and immune microenvironment remodeling. These findings establish a theoretical foundation for the development of novel diagnostic markers (such as serum snoRNAs) and targeted therapeutic approaches (e.g., inhibiting SNHG12 or SNORA38B).

#### 2.1.6. Breast Cancer

In recent years, the regulatory roles of snoRNAs and SNHGs in breast cancer have become increasingly well-defined. SnoRNAs primarily influence breast cancer progression through epigenetic modifications, modulation of signaling pathways, and regulation of ribosome activity. For instance, SNORA71A stabilizes ROCK2 mRNA by interacting with G3BP1, promoting EMT and metastasis [106]. SNORD50A/B accelerates the ubiquitination and degradation of wild-type p53 by enhancing the TRIM21-GMPS interaction, thereby driving tumor growth [36]. U50A contributes to everolimus resistance by inhibiting the mTOR/c-Myc pathway, while its overexpression also prolongs mitosis and suppresses colony formation, highlighting its dual functions [107]. Additionally, DKC1 overexpression enhances ribosomal activity by upregulating SNORA67, promoting the early malignant transformation of breast cancer cells [108]. Dyskerin modulates nuclear hormone receptor signaling by regulating snoRTs containing the H/ACA box, altering the reliance of breast cancer cells on hormone therapy [109]. Clinically, snoRNAs such as SNORD16, SNORA73B, SCARNA4, and SNORD49B exhibit significant overexpression in plasma, making them potential non-invasive biomarkers for early breast cancer detection [110]. Moreover, combined expression patterns of SNORD93 and SNORA16A can serve as predictors for local metastasis and patient prognosis [111]. Of note, sdRNAs also participate in tumor regulation. Studies have shown that sdRNA-93 is highly expressed in breast cancer cell line MDA-MB-231, and its expression level is positively correlated with cellular invasiveness. It can target and regulate the expression of Pipox, a sarcosine metabolism-related protein. In clinical samples, sdRNA-93 is significantly overexpressed in 92.8% of Luminal B Her2+ tumors, suggesting its potential role in the malignant progression of this subtype [37]. Members of the SNHG family modulate the malignant phenotype of breast cancer through ceRNA mechanisms, exosome-mediated delivery, and phase separation. For instance, SNHG22 sponges miR-324-3p to upregulate SUDS3, thereby promoting proliferation and invasion in triple-negative breast cancer (TNBC) [35]. SNHG8 enhances TNBC migration via the miR-335-5p/PYGO2 axis [112]. SNHG1 accelerates breast cancer progression through miR-641/RRS1 axes [113]. Exosome-derived SNHG12 promotes angiogenesis via the PBRM1/MMP10 axis, driving tumor growth [114], while SNHG9 inhibits the Hippo pathway through phosphatidic acid-induced LATS1 liquid–liquid phase separation, enhancing invasiveness [115]. In summary, snoRNAs and SNHGs play central roles in breast cancer proliferation, metastasis, drug resistance, and angiogenesis through multifaceted mechanisms, including RNA stability regulation, signaling pathway interaction, and exosome-mediated communication. These insights not only reveal potential targets, such as plasma snoRNAs, for molecular profiling and non-invasive diagnosis but also offer strategies for targeted therapy, such as inhibiting SNHG1 or SNORD50A, and addressing drug resistance by targeting U50A.

#### 2.1.7. Urinary System Malignancies

1. Bladder cancer

In recent years, the regulatory roles of snoRNAs and SNHGs in bladder urothelial carcinoma have become increasingly evident. SnoRNAs have demonstrated clinical potential through the development of prognostic models. For instance, the SNORS signature, consisting of five snoRNAs (DESs), effectively predicts bladder cancer prognosis. Its expression is modulated by copy number variations and DNA methylation, and correlates with pathways such as “extracellular matrix” and “EMT,” offering a novel biomarker and prognostic tool for bladder cancer [116]. Members of the SNHG family influence bladder cancer progression through various mechanisms. In the ceRNA mechanism, SNHG14 enhances endothelial cell-specific molecule 1 expression by sponging miR-211-3p, thereby promoting bladder cancer cell proliferation, migration, and inhibiting apoptosis [117]. Regarding transcription and protein stability regulation, SNHG18 destabilizes c-Myc protein via the ubiquitin-proteasome pathway, upregulates p21 expression, induces G0-G1 phase cell cycle arrest, and inhibits proliferation [39]. Meanwhile, SNHG1 binds to the RNA-binding protein HUR, leading to USP8 mRNA degradation, thereby reducing PTEN protein stability and driving the malignant transformation of basal muscle-invasive bladder cancer [118]. For interactions between super enhancers and signaling pathways: SNHG15 super enhancers recruit the transcription factor FOSL1 to initiate their transcription, while promoting ADAM12 expression via the WNT/CTNNB1 pathway, thereby accelerating bladder cancer cell proliferation and metastasis [38]. In conclusion, snoRNAs and SNHGs are integral to the onset, proliferation, metastasis, and treatment resistance of bladder cancer, acting through multiple mechanisms, including the construction of prognostic models, ceRNA regulation, transcription factor interactions, and protein stability modulation. These insights not only offer new avenues for molecular classification and non-invasive prognostic assessment of bladder cancer (e.g., the SNORS model), but also suggest potential targeted therapies, such as inhibiting SNHG1 or targeting SNHG15-SEs.

2. Renal cell carcinoma

Recent advances have clarified the mechanisms of action of snoRNAs and SNHGs in renal cell carcinoma (RCC), highlighting their potential in disease diagnosis, prognostic evaluation, and targeted therapy. From a clinical perspective, snoRNAs demonstrate dual utility in non-invasive diagnosis and treatment stratification. For instance, the differential expression of SNORD99, SNORD22, SNORD26, and SNORA50C in urine-derived exosomes is notably distinct in patients with clear cell renal cell carcinoma (ccRCC). When integrated with risk factors like obesity and hypertension, a highly accurate diagnostic model can be developed, offering a novel non-invasive approach for early detection [119]. Furthermore, exploratory analysis from the phase III IMmotion151 trial revealed that metastatic RCC patients with distinct snoRNAs transcriptome profiles may exhibit increased sensitivity to atezolizumab combined with bevacizumab treatment, indicating their potential in guiding personalized treatment strategies [120]. Members of the SNHG family promote RCC progression through various molecular mechanisms: in the ceRNA pathway, SNHG4 enhances RUNX2 expression by sponging miR-204-5p, thereby stimulating RCC cell proliferation and invasion while inhibiting apoptosis. Its elevated expression correlates with lymph node metastasis and poor patient survival outcomes [40]. Translational regulation and signaling pathway activation: SNHG6 interacts with the RNA-binding protein YBX1 to enhance the translation of HIF1α, promoting tumor growth and metastasis in ccRCC. Its overexpression serves as an independent prognostic marker for poor outcomes [41]. In conclusion, snoRNAs and SNHGs are central to the early diagnosis, prognosis prediction, and targeted treatment of RCC through biomarker identification (e.g., urinary exosomal snoRNAs) and molecular pathway regulation (e.g., the SNHG4/miR-204-5p/RUNX2 axis and the SNHG6/YBX1/HIF1α pathway). These observations establish a foundation for developing precision diagnostic and therapeutic tools based on non-coding RNAs and introduce new molecular targets, such as SNHG6, for combined immunotherapy or targeted interventions. Further research is required to validate the clinical utility of these markers and to explore their interactions with other signaling pathways, optimizing treatment strategies.

3. Prostate cancer

Recent studies have increasingly highlighted the regulatory roles of SNHG family members in prostate cancer, where they drive disease progression via multiple pathways, including ceRNA interactions, transcriptional regulation, and chemoresistance. SNHGs promote prostate cancer development through mechanisms such as chemoresistance and ceRNA regulation. For example, SNHG6 relieves target gene inhibition by sponging miR-186 and upregulating multidrug resistance proteins (e.g., MRP1, MDR1), thereby enhancing paclitaxel resistance [43]. SNHG11 promotes cell proliferation and metastasis through the miR-184/IGF-1R axis, with high expression levels correlating with drug resistance and poor prognosis [121]. Regarding transcription factor-driven and signaling pathway activation, SNHG4 is overexpressed under the regulation of SP1, promoting prostate cancer cell proliferation, migration, and invasion by sponging miR-377 to upregulate the oncogene ZIC5. Its expression is significantly associated with tumor stage and shortened survival [42]. Additionally, SNHG12 accelerates tumorigenesis by targeting miR-133b, and its elevated expression serves as an independent prognostic marker [122]. Regarding diagnostic and prognostic stratification potential, SNHG25, which is highly expressed in prostate cancer, correlates with Gleason score, lymph node metastasis, and shortened progression-free survival, demonstrating diagnostic efficacy (AUC = 0.923) and independent prognostic value, thereby establishing a foundation for clinical stratification [123]. Beyond SNHGs, sdRNAs also contribute to prostate cancer pathogenesis. Specifically, sdRNA-D19b and sdRNA-A24 are significantly upregulated in prostate cancer tissues, where they enhance PC3 cell proliferation, migration, and chemoresistance—sdRNA-D19b to paclitaxel and sdRNA-A24 to dasatinib—by targeting tumor suppressors CD44 and CDK12, respectively. This outlines a novel sdRNA-mediated mechanism that promotes aggressive phenotypes and treatment resistance in castration-resistant prostate cancer (CRPC) [44]. Although research on snoRNAs remains limited, their production is often hosted within SNHG transcripts, suggesting a broader regulatory network where SNHGs may modulate sdRNA activity. These findings underscore the multifaceted roles of non-coding RNAs in prostate cancer and highlight the potential of targeting specific SNHG-sdRNA axes for therapeutic intervention, such as disrupting the SNHG4/SP1 or SNHG11/IGF-1R pathways. Future studies should explore the synergistic interactions between SNHGs and sdRNAs, as well as their utility in liquid biopsies for improving precision diagnostics and treatments.

#### 2.1.8. Gynecological Malignancies

1. Cervical cancer

In recent years, the regulatory functions of snoRNAs and SNHGs in cervical cancer have been increasingly elucidated. They influence disease progression and therapeutic resistance by modulating key protein stability and signaling pathways. snoRNAs promote cervical cancer progression by targeting tumor suppressors. For instance, SNORD6 is overexpressed in cervical cancer tissues, inhibiting apoptosis and driving tumor growth by facilitating E6-mediated p53 ubiquitination and degradation. Silencing SNORD6 in animal models markedly reduces tumor volume, indicating its potential as a therapeutic target [45]. Members of the SNHG family regulate radiosensitivity through the ceRNA mechanism; SNHG6 sponges miR-485-3p, releasing STYX protein and thereby enhancing cervical cancer cell resistance to radiotherapy. Its silencing inhibits cell proliferation and increases radiosensitivity [46]. In summary, snoRNAs and SNHGs are central regulators of cervical cancer proliferation, apoptosis, and radioresistance through the modulation of p53 stability (e.g., SNORD6) and ceRNA-mediated signaling pathways (e.g., SNHG6/miR-485-3p/STYX axis, SNHG12/miR-148a/CDK1 pathway). These results not only offer a novel perspective on the molecular mechanisms of cervical cancer but also provide a theoretical foundation for the development of precision therapies targeting non-coding RNAs (e.g., inhibiting SNORD6 or SNHG12), particularly in overcoming radioresistance. Further investigation into the synergistic roles of snoRNAs and SNHGs, as well as their potential in clinical diagnosis, is warranted.

2. Endometrial cancer

In recent years, the regulatory roles of snoRNAs and SNHGs in endometrial cancer have become well-established. These RNAs significantly contribute to tumorigenesis, metastasis, and treatment resistance via RNA modification and ceRNA mechanisms. SnoRNAs regulate critical target genes through epigenetic modifications. For example, SNORD89 inhibits the translation of the pro-apoptotic protein Bim through 2′-*O*-methylation, thereby reducing its expression and promoting the proliferation and migration of endometrial cancer cells. Elevated SNORD89 expression correlates strongly with lymph node metastasis [124]. SNORD104 interacts with the methyltransferase FBL, enhancing 2′-*O*-methylation of PARP1 mRNA, which stabilizes the mRNA and promotes its nuclear localization, ultimately driving tumor growth [47]. SNHG family members promote tumor progression through ceRNA mechanisms and interactions with signaling pathways. For instance, SNHG25 sponges miR-497-5p, relieving its suppression of fatty acid synthase, thereby promoting cancer cell proliferation, migration, and inhibiting apoptosis. High SNHG25 expression is associated with poor patient prognosis, highlighting its potential as a therapeutic target [48]. SLERT, a long non-coding RNA that ends with an H/ACA snoRNA, interacts with the methyltransferase METTL3 to enhance m^6^A modification of brain-derived neurotrophic factor (BDNF) mRNA, thereby stabilizing it and activating the BDNF/TRKB signaling pathway. This process induces EMT and lung metastasis. Elevated expression levels of SLERT are observed in both tissue and plasma, making it a potential biomarker for diagnosis and prognosis [125]. In summary, snoRNAs and SNHGs are integral to the proliferation, metastasis, and metabolic reprogramming of endometrial cancer through RNA modifications (e.g., methylation, m^6^A modification) and ceRNA-mediated signaling pathways, such as the SNHG25/miR-497-5p/FASN axis and the SLERT/BDNF/TRKB pathway. These insights offer novel strategies for targeting RNA-modifying enzymes (e.g., FBL, METTL3) and key signaling nodes (e.g., PARP1, BDNF), while also providing a theoretical framework for developing non-coding RNA-based diagnostic and therapeutic approaches, including the inhibition of SNHG25 or targeting SLERT. Future research should focus on further elucidating the synergistic regulatory networks of snoRNAs and SNHGs, as well as exploring their potential in liquid biopsy applications (e.g., plasma exosome detection) to advance the precise diagnosis and treatment of endometrial cancer.

3. Ovarian cancer

In recent years, SNHGs, a key member of the lncRNAs, have demonstrated multifaceted regulatory effects in ovarian cancer, influencing chemotherapy resistance, invasion, metastasis, and prognosis through the ceRNA mechanism. Research indicates that SNHG5 sponges miR-23a, relieving its inhibition of downstream target genes, which significantly enhances ovarian cancer cell sensitivity to paclitaxel [49]. In contrast, high expression of SNHG1 contributes to chemotherapy resistance by suppressing miR-216b-5p activity, correlating with poor patient prognosis [126]. Regarding tumor invasion and metastasis, SNHG20 activates the Rho kinase signaling pathway by targeting the miR-148a/ROCK1 axis, promoting ovarian cancer cell migration and invasion [127]. SNHG6 upregulates the oncogene YAP1 by inhibiting miR-543 expression, driving EMT and malignant phenotype [128]. Furthermore, SNHG10 enhances the expression of the tumor suppressor gene BIN1 by sponging miR-200a-3p, effectively inhibiting ovarian cancer cell proliferation, migration, and EMT. Its reduced expression is closely linked to poor prognosis [50]. These studies not only highlight the dual tumor-promoting and tumor-suppressing roles of SNHGs in ovarian cancer but also emphasize their potential as biomarkers or therapeutic targets, laying a crucial theoretical foundation for the development of precision treatment strategies targeting the SNHG/miRNA axis.

#### 2.1.9. Hematological Malignancies

The progression of leukemia is intricately linked to the multi-level regulation of snoRNAs and SNHGs. SnoRNAs contribute to leukemia development through both classical and non-classical mechanisms: SNORD42A mediates 2′-*O*-methylation of 18S rRNA, sustaining the proliferation of acute myeloid leukemia cells [51]; SNORD118 and SNORD3A specifically regulate leukemia cell survival through chromatin interactions [129]; and the snoRNA-associated lncRNA LNC-SNO49AB promotes malignant transformation by activating the RNA editing function of ADAR1 [130]. In contrast, SNHGs function as ceRNAs, sponging miRNAs to modulate key signaling pathways. For instance, SNHG5 enhances chemotherapy resistance through the miR-32/DNAJB9 axis [131], and promotes angiogenesis via the YY1-mediated miR-26b/CTGF/VEGFA pathway [132]; SNHG1 and SNHG6 inhibit cell growth or enhance differentiation sensitivity through the miR-489-3p/SOX12/Wnt/β-catenin pathway, respectively [52,133]. These studies reveal that snoRNAs directly influence leukemia through epigenetic modifications and RNA editing, while SNHGs integrate miRNAs and downstream target genes within a complex ceRNA network, collectively forming a multidimensional regulatory framework for leukemia initiation and progression. Interventions targeting snoRNAs (e.g., SNORD42A, LNC-SNO49AB) or SNHGs may offer novel strategies to overcome drug resistance and inhibit leukemia progression by disrupting RNA modification, editing, or key signaling pathways.

##### Hallmarks of snoRNA-Mediated Cancer Regulation

The functional impact of snoRNAs in oncology is not isolated; rather, it is intricately interwoven with the core signaling pathways that drive malignant transformation. In this signaling crosstalk, snoRNAs act as both downstream effectors and upstream regulators, interacting especially with the p53, PI3K/AKT/mTOR, and NF-κB networks. The p53 network is a central hub for these interactions. This relationship is multifaceted: on one hand, oncogenic snoRNAs such as SNORD17 and SNORA42 act as disruptors of the p53 pathway [134,135]. For example, SNORD17 non-canonically anchors key p53 regulators (like NPM1 and MYBBP1A) in the nucleolus, thereby inhibiting p53 pathway activity [134]; on the other hand, other snoRNAs (like SNORA13) can serve as ‘sensors’ for p53 activation. SNORA13 non-canonically interacts with the ribosomal protein RPL23 to mimic a state of nucleolar stress, which in turn triggers p53-mediated cellular senescence [136,137]. Furthermore, many tumor-suppressive snoRNAs (such as SNORD44) are themselves downstream effectors of p53, mediating p53-dependent or -independent apoptosis [62]. Beyond the p53 pathway, snoRNA biosynthesis is largely controlled by the pro-growth PI3K/AKT/mTOR pathway, which is commonly activated in cancer [138]. This integration also presents therapeutic opportunities, as snoRNAs can regulate key downstream effectors of mTOR. For instance, SNORA23 can inhibit the phosphorylation of 4EBP1, a target that is poorly inhibited by mTOR inhibitors like Rapamycin. Consequently, the combined use of SNORA23 mimetics and Rapamycin could synergistically block both major downstream branches of mTORC1 (RPS6 and 4EBP1) [139]. Similarly, SNORD44 has also demonstrated a synergistic anti-tumor effect with Rapamycin [62]. Finally, snoRNAs have also been shown to directly activate other oncogenic pathways. The pro-oncogenic snoRNA SNORA42 activates the NF-κB signaling pathway by mediating the interaction between the DHX9 and p65 subunits, leading to the upregulation of downstream target genes (including MMP2, MMP9, VEGF, and BCL2) and thereby driving tumor proliferation, invasion, and angiogenesis [135].

snoRNA function in cancer is highly context-dependent, demonstrating a dual capacity to act as both oncogenes and tumor suppressors. On the pro-oncogenic side, SNORA42 is a well-documented, multi-faceted oncogene found to be highly expressed in CRC and NSCLC. In CRC, high SNORA42 expression is an independent prognostic indicator, significantly associated with worse overall survival (OS) (HR: 2.11) and disease-free survival (DFS) (HR: 3.17). Its mechanisms include promoting proliferation and invasion by activating the NF-κB pathway and inhibiting the p53 pathway, as well as maintaining cancer stem cell (CSC) stemness in NSCLC by preserving levels of core transcription factors like OCT4, Nanog, and Sox2 [140,141]. Conversely, many snoRNAs serve critical tumor-suppressive roles. SNORD44 (also from the GAS5 host gene) is consistently downregulated in CRC, where its overexpression inhibits tumor growth by inducing caspase-dependent apoptosis [142]. Likewise, SNORA52 is significantly downregulated in HCC and serves as an independent predictor of poor prognosis, with its suppressor function presumed to be linked to its classical role in maintaining normal ribosome function [143,144]. The tissue-specific paradox of snoRNA function is best exemplified by SNORA23. In pancreatic ductal adenocarcinoma (PDAC), SNORA23 acts as an oncogene, where it is upregulated and promotes invasion by non-canonically increasing SYNE2 protein expression [145]. In stark contrast, SNORA23 is a definitive tumor suppressor in HCC. In HCC, it exerts its anti-tumor effects via a dual mechanism: classically, by impairing 28S rRNA methylation, and non-classically, by inhibiting the phosphorylation of the mTOR pathway effector 4EBP1 [139]. This “dual-agent” role highlights that snoRNA function is not fixed but is determined by the cellular context and the availability of downstream interaction partners.

## 3. Mechanisms of snoRNAs and SNHGs

**Figure 1:**
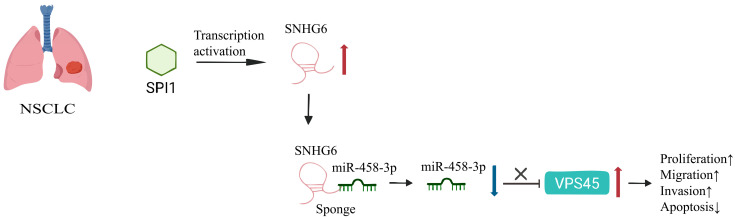
SPI1-driven SNHG6 upregulation sponges miR-485-3p in NSCLC, derepressing its target VPS45. Elevated VPS45 promotes tumor progression by enhancing proliferation, migration, and invasion while suppressing apoptosis.

The biological functions of snoRNAs and SNHGs are mainly exerted through the following four mechanisms.

(I) ceRNA Regulatory Network (Figure 1)

**Figure 2:**
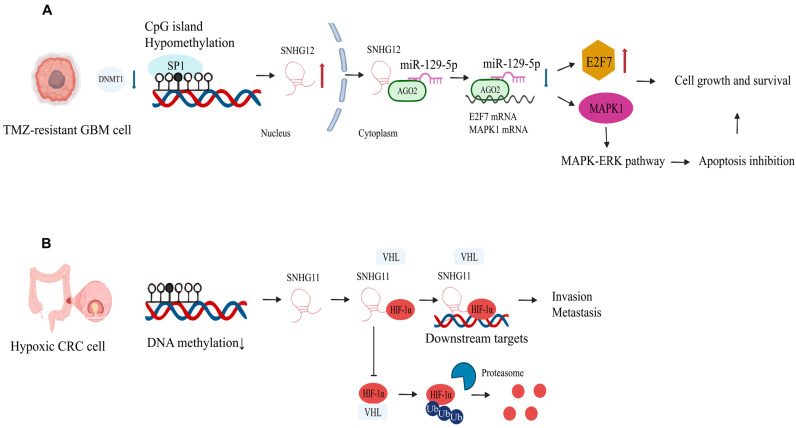
(**A**) This mechanistic scheme illustrates the molecular mechanism by which hypomethylation of the SNHG12 promoter drives temozolomide (TMZ) resistance in glioblastoma (GBM). (**B**) In hypoxic CRC cells, DNA demethylation upregulates SNHG11, which binds and stabilizes HIF-1α by blocking VHL-mediated ubiquitination and proteasomal degradation, activating metastasis-associated genes to drive invasion.

SNHGs, as key components of the ceRNA regulatory network, play a significant role in the progression of various cancers. In prostate cancer, SNHG6 modulates cancer cell sensitivity to paclitaxel by sponging miR-186 [43], SNHG12 influences tumorigenesis through targeting miR-133b [122], and SNHG4 enhances ZIC5-mediated tumor growth and metastasis via the regulation of miR-377 [42]. In NSCLC, SNHG6 acts through the miR-485-3p/VPS45 axis [146], while SNHG15 exerts its effects via the miR-451/MDR-1 axis [102]. In TNBC, SNHG22 drives tumor progression by sponging miR-324-3p and upregulating SUDS382; LRRC75A-AS1 (SNHG29) promotes tumor progression by targeting the miR-380-3p/BAALC pathway [35,147], and SNHG8 promotes tumor progression through the miR-335-5p/PYGO2 axis [112]. Additionally, in various cancers, including ovarian cancer [49,127,148], colorectal cancer [27,28,85], and neuroblastoma [17,18], distinct lncRNAs such as SNHG5, SNHG17, and SNHG16 form ceRNA networks by interacting with specific miRNAs, thereby regulating key aspects of tumor cell behavior, including proliferation, migration, invasion, apoptosis, and drug resistance. These ceRNA networks hold promise as potential targets for cancer therapy.

(II) Epigenetic regulation (Figure 2)

**Figure 3:**
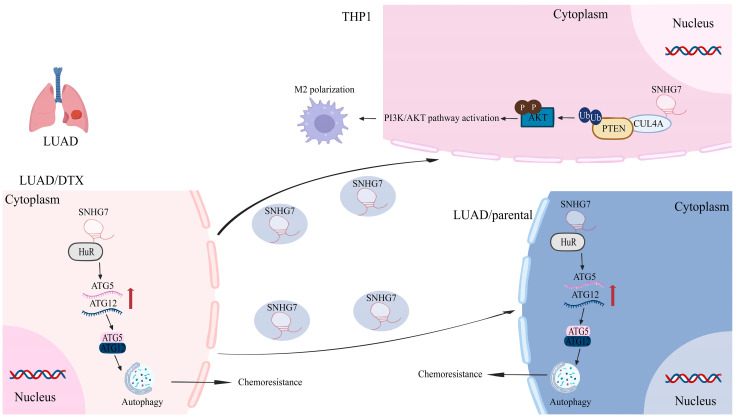
Exosomal SNHG7 drives docetaxel resistance and M2 macrophage polarization in LUAD. Resistant LUAD cells transfer exosomal SNHG7 to parental cells, boosting chemoresistance. SNHG7 recruits HuR to stabilize ATG5/ATG12 mRNAs, activating autophagy and drug resistance. Exosomal SNHG7 internalized by macrophages recruits CUL4A, triggering PTEN ubiquitination/degradation. PTEN loss activates PI3K/AKT, driving pro-tumor M2 polarization.

DNA methylation regulates SNHG expression. In GBM, the SNHG12 promoter exhibits abnormally low DNA methylation, increasing its susceptibility to Sp1 transcription factor binding, which subsequently modulates SNHG12 expression. Upregulation of SNHG12 occurs in temozolomide-resistant cells and tissues, where its overexpression drives acquired resistance to temozolomide, while SNHG12 knockdown restores drug sensitivity [16]. In colorectal cancer, promoter hypomethylation leads to the upregulation of SNHG11, which correlates with poor prognosis in affected patients [83]. Histone modifications also influence SNHG expression. SNHG14 is highly expressed in HCC tissues and cells, where it promotes polyadenylate-binding protein 1 upregulation via H3K27 acetylation, enhancing cell proliferation, migration, and angiogenesis [149]. SNHGs interact with transcription factors; for example, SP1 binds to the SNHG4 promoter to induce its upregulation [42], while YY1 trans-activates the SNHG5 promoter, leading to increased SNHG5 expression in acute myeloid leukemia [132].

(III) Exosome-mediated intercellular communication (Figure 3)

**Figure 4:**
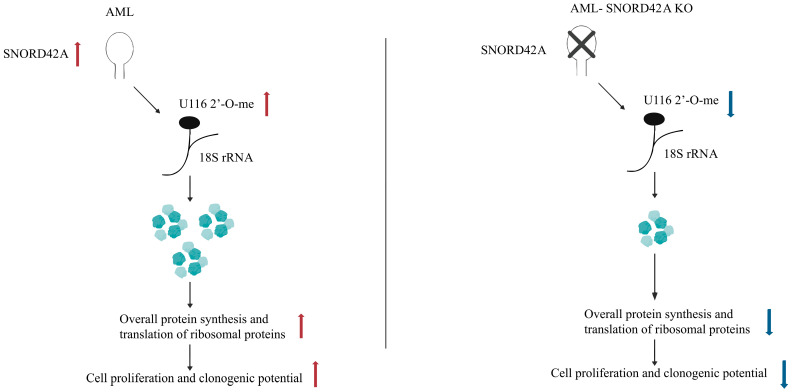
This mechanistic scheme illustrates SNORD42A-mediated 2′-*O*-methylation at U116 on 18S rRNA as critical for AML proliferation: its presence enhances ribosomal translation and protein synthesis, sustaining clonogenicity, while knockout disrupts methylation, impairing ribosome function and suppressing growth.

Elevated expression of SNHG7 is observed in docetaxel-resistant cells in lung adenocarcinoma with resistance to docetaxel. Furthermore, exosomal SNHG7 is shown to be transferred from docetaxel-resistant lung adenocarcinoma cells to their parental counterparts. Mechanistically, SNHG7 promotes autophagy by recruiting human antigen R (HuR), which stabilizes autophagy-related genes ATG5 and ATG12. Additionally, SNHG7 interacts with CUL4A to promote the ubiquitination and degradation of PTEN, thereby activating the PI3K/AKT pathway, inducing M2 macrophage polarization, and ultimately enhancing docetaxel resistance in lung adenocarcinoma cells [103]. In HCC, SNHG16 is significantly upregulated in both tumor tissues and cell lines. Exposure of human umbilical vein endothelial cells to exosomes from HCC cells containing SNHG16 results in markedly increased proliferation, migration, and angiogenesis [94].

(IV) Ribosomal RNA modification and translational regulation (Figure 4)

SNORD42A, a C/D box snoRNA, directs the 2′-*O*-methylation of uridine 116 in 18S rRNA. Deletion of SNORD42A results in decreased 2′-*O*-methylation at 18S-U116, which is linked to a specific reduction in ribosomal protein synthesis. Consequently, leukemia cells lacking SNORD42A exhibit reduced cell size. This suggests that SNORD42A-mediated rRNA modification modulates translation efficiency, thereby influencing the proliferation of acute myeloid leukemia cells, illustrating the mechanism by which SNHGs impact translation through snoRNA-mediated rRNA modification [51].

## 4. Clinical Application Potential of snoRNAs and SNHGs

(I) Diagnostic and prognostic markers

Recent pan-cancer studies have established that snoRNAs and their derived sdRNAs are a prevalent class of noncoding RNA biomarkers with significant implications for cancer immunity and patient survival. SNORA56 is upregulated in both colorectal cancer tissues and plasma, correlating with patient prognosis. Plasma levels of SNORA56 enable non-invasive detection and prognostic assessment of colorectal cancer, offering valuable insights for clinical diagnosis and treatment [75]. In RCC, SNORD15A expression is significantly elevated in tumor tissues compared to adjacent normal tissues. Its levels are also increased in the urine sediment of RCC and early RCC patients relative to healthy controls, suggesting the potential of urine sediment-based SNORD15A detection for non-invasive RCC diagnosis. The combination of three snoRNAs—SNORD15A, SNORD35B, and SNORD60—effectively differentiates RCC and early RCC from healthy individuals. Analysis using receiver operating characteristic curves demonstrates that this combination enhances diagnostic specificity and accuracy compared to using a single RNA marker [150]. Collectively, the mapping of the pan-cancer sdRNAome provides definitive evidence that snoRNAs and their derivative sdRNAs are potent markers, with expression signatures that distinguish cancer types, reflect the immune microenvironment, and predict patient survival [151].

(II) Therapeutic targets

In tumor therapeutics, emerging strategies targeting various cancers have shown promise. For endometrial cancer, antisense oligonucleotides (ASO) targeting C/D box snoRNA 89 (SNORD89) have been explored. Due to its elevated expression in cancer tissues, silencing SNORD89 suppresses cancer cell proliferation and migration. ASO-SNORD89 shows potential as an effective therapeutic approach [124].

## 5. Summary and Prospect

SnoRNAs and SNHGs are crucial to tumor initiation and progression. SnoRNAs drive tumor progression through RNA modification and involvement in various signaling pathways, while SNHGs influence tumor behavior via ceRNA networks and epigenetic mechanisms, impacting cell proliferation, metastasis, drug resistance, and patient prognosis. Certain snoRNAs and SNHGs hold promise as diagnostic and prognostic biomarkers and potential therapeutic targets. For instance, plasma-derived snoRNAs offer a non-invasive approach for diagnosis, and interventions targeting specific snoRNAs or SNHGs have demonstrated therapeutic benefits in some studies. Future efforts should focus on thoroughly mapping the dynamic regulatory networks of snoRNAs and SNHGs within tumors, elucidating their synergistic interactions. Additionally, further exploration of their role in tumor diagnosis is essential to enhance early diagnostic accuracy. Strengthening clinical translational research is crucial to validating the efficacy of current biomarkers and therapeutic targets, developing targeted treatments, and advancing integrated therapeutic strategies based on snoRNAs and SNHGs. This will support the advancement of precision oncology, offering novel approaches for tumor management.

Despite significant progress in the snoRNA field, several key challenges and future directions remain. First, a deeper mechanistic understanding is required to decipher the “oncoribosome code”—specifically, how snoRNA-mediated rRNA modification profiles precisely determine the preferential translation of specific oncogene mRNAs. Second, translating these discoveries into clinical practice faces a major hurdle in therapeutic delivery. While preclinical models using antisense oligonucleotides (ASOs) to target oncogenic snoRNAs have shown promise, developing systems for efficient and specific in vivo delivery of these RNA therapeutics, especially to solid tumors, remains a significant challenge. Finally, a critical emerging frontier is the intersection of snoRNAs with the tumor immune microenvironment. Preliminary evidence indicates that the mTOR pathway, which dominates snoRNA regulation, is also a key regulator of immune T-cell function. Furthermore, specific snoRNAs have been correlated with the abundance of tumor-infiltrating NK cells and CD8+ T cells. This raises urgent questions about whether snoRNAs mediate immune crosstalk and whether targeting them could synergize with immune checkpoint inhibitors, representing a highly promising avenue for future research.

## Figures and Tables

**Table 1:** Functions of snoRNAs and SNHGs in Various Types of Malignancies.

Types of Cancer	Expression in Cancer	Expression	Regulatory Site	Function	Ref.
Neurologic malignancies					
Glioblastoma	U3 snoRNA	Upregulated	ZBTB7A	Enhances aerobic glycolysis, proliferative capacity	[14]
		Upregulated	TRIM24/DNA-PKcs	induces epigenetic remodeling and epithelioid transformation	[15]
	SNHG12	Upregulated	miR-129-5p/MAPK1/E2F7	Activates MAPK/ERK cascade, drives cell cycle progression, promotes temozolomide resistance	[16]
Neuroblastoma	SNHG7	Upregulated	miR-323a-5p/miR-342-5p; CCND1	Enhances migration, invasion, glycolytic activity	[17]
	SNHG16	Upregulated	miR-338-3p/PLK4; PI3K/AKT pathway	Promotes cisplatin resistance	[18]
Bone malignancies	SNHG6	Upregulated	EZH2/KLF6/SP1	Promotes H3K27me3-mediated silencing of KLF6, drives proliferation, migration, and invasion	[19]
Head and neck squamous cell carcinoma	SNHG3	Downregulated	RPS27A/RPL8; ribosome biogenesis	Drives tumor progression via metabolic remodeling, immune regulation, and TME modulation	[20]
Digestive system malignancies					
Esophageal cancer					
	SNHG12	Upregulated	miR-6835-3p/BMI1; IGF2BP2/CTNNB1	Induces EMT, reinforces stem-like properties, accelerates metastasis	[21]
	SNHG17	Overexpressed	miR-338-3p/SOX4	Promotes cellular proliferation and invasion	[22]
Gastric cancer					
	SNHG12	Upregulated	miR-218-5p/YWHAZ/β-catenin	Promotes metastasis and EMT	[23]
		Overexpressed	miR-129-5p/E2F7/MAPK/ERK	Promotes peritoneal mesothelial cell apoptosis	[24]
	SNHG22	Upregulated	EZH2; miR-200c-3p/Notch1	Enhances proliferation, invasion; silences tumor suppressors, upregulates Notch1	[25]
Colorectal cancer					
	SNORA24	Upregulated	p53 protein (proteasome pathway)	Promotes proliferation	[26]
	SNHG17	Upregulated	miR-23a-3p/CXCL12	Promotes proliferation and migration	[27]
		Upregulated	Trim23-PES1/miR-339-5p-FOSL2	Promotes proliferation and metastasis	[28]
Hepatocellular Carcinoma					
	SNORD52	Upregulated	CDK1	Promotes cell cycle progression and tumor growth	[29]
	SNHG1	Upregulated	SND1/m6A/SLC7A11	Sorafenib resistance	[30]
Pancreatic cancer					
	SNHG6	Upregulated	miR-26a-5p/FUBP1/EMT markers	Promotes EMT (↑N-cadherin, vimentin, β-catenin; ↓E-cadherin), proliferation, and metastasis	[31]
	RRP9 (U3 snoRNA binder)	Upregulated	IGF2BP1/AKT signaling	Enhances DNA repair, inhibits apoptosis, induces gemcitabine resistance	[32]
Lung cancer					
	SNORA65, SNORA7A/B	Upregulated	Ribosomal pseudouridylation	Promotes proliferation/metastasis	[33]
	SNHG11	Upregulated	miR-4436a/CTNNB1, activates Wnt/β-catenin	Promotes proliferation, migration, invasion, and EMT	[34]
Breast cancer					
	SNHG22	Upregulated	miR-324-3p/SUDS3	Promotes proliferation and invasion	[35]
	SNORD50A/B	Downregulated	TRIM21-GMPS/p53	Accelerates p53 degradation, drives tumor growth	[36]
	sdRNA-93	Upregulated	Pipox 3′UTR	Enhances cellular invasion; specifically overexpressed in Luminal B Her2+ subtype	[37]
Urinary system malignancies					
Bladder cancer					
	SNHG15	Upregulated	FOSL1/ADAM12; WNT/CTNNB1 pathway	Accelerates proliferation and metastasis	[38]
	SNHG18	Downregulated	c-Myc/p21	Induces G0-G1 arrest, inhibits proliferation	[39]
Renal cell carcinoma					
	SNHG4	Upregulated	miR-204-5p/RUNX2	Stimulates proliferation, invasion; inhibits apoptosis.	[40]
	SNHG6	Upregulated	YBX1/HIF1α translation	Promotes tumor growth, metastasis.	[41]
Prostate cancer					
	SNHG4	Upregulated	SP1/miR-377/ZIC5	Drives proliferation, migration, invasion	[42]
	SNHG6	Upregulated	miR-186/MRP1/MDR1	Enhances paclitaxel resistance by upregulating multidrug resistance proteins	[43]
	sdRNA-D19b	Upregulated	CD44	Promotes cell proliferation, migration, and paclitaxel resistance	[44]
	sdRNA-A24	Upregulated	CDK12	Promotes cell proliferation and dasatinib resistance	[44]
Gynecological malignancies					
Cervical cancer					
	SNORD6	Upregulated	E6-mediated p53 ubiquitination	Inhibits apoptosis, drives tumor growth	[45]
	SNHG6	Upregulated	miR-485-3p/STYX	Enhances radioresistance	[46]
Endometrial cancer					
	SNORD104	Upregulated	PARP1 mRNA (2′-*O*-methylation)	Stabilizes PARP1 mRNA, drives tumor growth	[47]
	SNHG25	Upregulated	miR-497-5p/fatty acid synthase	Promotes proliferation, migration; inhibits apoptosis	[48]
Ovarian cancer					
	SNHG5	Downregulated	miR-23a/downstream targets	Enhances paclitaxel sensitivity	[49]
	SNHG10	Downregulated	miR-200a-3p/BIN1	Inhibits proliferation, migration, EMT	[50]
Hematological malignancies					
	SNORD42A	Upregulated	Ribosomal RNA modification	Sustains proliferation of acute myeloid leukemia (AML) cells	[51]
	SNHG1	Upregulated	miR-489-3p/SOX12/Wnt/β-catenin pathway	Suppresses cell growth	[52]

**Table 2:** Contrasting Oncogenic and Tumor-Suppressive Roles of Key snoRNAs in Human Cancers.

snoRNA	Classification	Canonical Nucleolar Function	Disease Associations	Cancer Type(s)	Expression in Cancer	Ref.
SNORA42	H/ACA Box	rRNA pseudouridylation	Oncogene: Promotes proliferation, migration, invasion; inhibits apoptosis. High expression correlates with poor prognosis.	Non-Small Cell Lung Cancer (NSCLC)	Upregulated	[60]
SNORD44	C/D Box	rRNA 2′-*O*-ribose methylation	Context-dependent role: In Prostate Cancer: Upregulated with its sdRNAs during malignant transformation. In Colorectal Cancer: Acts as a tumor suppressor in concert with its host gene GAS5.	Prostate Cancer (PCa), Colorectal Cancer (CRC)	PCa: Upregulated CRC: Downregulated	[61,62]
SNORA21	H/ACA Box	rRNA pseudouridylation	Dual Role, highly context-dependent: In Gastric Cancer: Acts as an oncogene; high expression linked to metastasis and poor survival. In Gallbladder Cancer: Functions as a tumor suppressor; overexpression inhibits tumorigenesis.	Gastric Cancer (GC), Gallbladder Cancer (GBC)	GC: Upregulated GBC: Downregulated	[63,64]

## Data Availability

Not applicable.
